# Value of Fast MVO Identification From Contrast-Enhanced Cine (CE-SSFP) Combined With Myocardial Strain in Predicting Adverse Events in Patients After ST-Elevation Myocardial Infarction

**DOI:** 10.3389/fcvm.2021.804020

**Published:** 2022-02-21

**Authors:** Min Zhang, Yuan Lu, Zhi Li, Yameng Shao, Lei Chen, Yu Yang, Jianning Xi, Minglong Chen, Tingbo Jiang

**Affiliations:** ^1^Department of Cardiology, The First Affiliated Hospital of Soochow University, Suzhou, China; ^2^Department of Cardiology, The Affiliated Hospital of Xuzhou Medical University, Xuzhou, China; ^3^Department of Radiology, The Affiliated Hospital of Xuzhou Medical University, Xuzhou, China; ^4^Department of Cardiology, The First Affiliated Hospital of Nanjing Medical University, Nanjing, China

**Keywords:** microvascular obstruction, myocardial strain, myocardial infarction, cardiac magnetic resonance, contrast-enhanced steady-state free precession

## Abstract

**Objectives:**

Cardiac magnetic resonance imaging (CMR) can be used for a one-step evaluation of myocardial function and pathological features after acute ST-elevation myocardial infarction (STEMI). We aimed to evaluate the value of fast microvascular occlusion (MVO) identification from contrast-enhanced steady-state free precession (CE-SSFP) combined with myocardial strain in predicting major cardiovascular adverse events (MACEs) in primary percutaneous coronary intervention (pPCI) patients with STEMI.

**Methods:**

In total, 237 patients with STEMI who received pPCI and completed CMR scans within the following week were enrolled, MVO identification and the myocardial strain analysis were performed in CE-SSFP images without an additional method. The primary endpoint was the presence of MACE, which is defined as a composite of death, reinfarction, and congestive heart failure (HF).

**Results:**

After 13 months of follow-up [interquartile range (IQR): 11–24], 30 patients (14%) developed MACE; the MVO (hazard ratio (HR) was 3.10; 95% CI: 1.14–8.99, *p* = 0.028), and the infarct size (IS) (HR: 1.03; 95% CI: 1.0–1.06, *p* = 0.042) and global longitudinal strain (GLS) (HR: 1.08; 95% CI: 1.01–1.17, *p* = 0.029) were finally associated with MACE. Receiver operating characteristic (ROC) analyses show that the area under the curve (AUC) of GLS was large (0.73 [95% CI, 0.63–0.82], *p* = 0.001), and the best cut-off was −11.8%, with 76.7% sensitivity and 58.9% specificity, which are greater than those of IS (0.70, 95% CI, 0.60–0.81, *p* < 0.001) and MVO (0.68, 95% CI, 0.58–0.78, *p* < 0.001). However, combining MVO and GLS resulted in a much greater finding (AUC = 0.775, 95% CI: 0.727–0.824, *p* < 0.001) and better sensitivity and specificity (83.3%, 66.5%).

**Conclusion:**

Microvascular occlusion identification from contrast-enhanced cine combined with myocardial strain could be a quick and reliable option for patients with STEMI who underwent pPCI; it correlates well with the prognosis of patients with STEMI in terms of MACE.

## Introduction

Myocardial infarction mortality declines with the growing popularity of primary percutaneous coronary intervention (pPCI) and advanced management. However, the reperfusion process can aggravate the myocardial injury and cardiomyocyte death, a phenomenon called “myocardial reperfusion injury” (1), contributing to microvascular occlusion (MVO). MVO and subsequent intramyocardial hemorrhage (IMH) are strongly associated with mortality and hospitalization for heart failure (HF) (2). Therefore, effective identification of MVO and early risk assessment are recommended to reduce complications post-myocardial infarction (MI), such as new-onset HF.

As a new non-invasive imaging technology in patients with ST-elevation myocardial infarction (STEMI), cardiac magnetic resonance imaging (CMR) can evaluate cardiac function and accurately describe the myocardial injury and infarct pathology (infarct size [IS] and MVO) in one step (3), providing considerable prognostic information over established clinical parameters and traditional left ventricular ejection fraction (LVEF) (4, 5). Cine with balanced steady-state free precession (b-SSFP) is a regular part of each CMR protocol and has the advantages of high speed, superior signal-to-noise ratio, and short breath-holding and can provide sufficient information to assess cardiac function markers more comprehensively, such as the myocardial strain.

Cardiac magnetic resonance imaging feature-tracking (FT) myocardial strain analysis has increasingly been used to detect local and subtle myocardial dysfunction in various cardiovascular diseases, such as myocardial infarction (6, 7). It is a more in-depth assessment of myocardial deformation in three different directions, which correspond to the geometry of myocardial fibers. At present, FT myocardial strain involves retrospective motion tracking of steady-state free precession cine images, has become an advanced measurement of LV performance, a valuable tool to optimize post-infarction risk assessment (8, 9), and a potential supplement to LVEF for assessment in patients with STEMI. This study identified MVO from contrast-enhanced cine (CE-SSFP) while performing the FT strain analysis and attempted to evaluate the predictive value of rapid MVO identification in combination with myocardial strain in adverse events in patients with post-STEMI.

## Methods

### Participant Population

Between September 2019 and September 2020, we selected 237 patients who first developed STEMI and received pPCI. All patients had informed consent. The Ethics Committee approved the study of the Affiliated Hospital of Xuzhou Medical University. The criteria for inclusion were as follows: first STEMI meeting the European Society of Cardiology/the American College of Cardiology/the American Heart Association (ESC/ACC/AHA) committee criteria (8), revascularization by pPCI within 24 h of ischemic symptoms, and Killip class <3 during CMR scan. The exclusion criteria were as follows: the previous history of myocardial infarction or coronary artery bypass grafting; severe renal insufficiency; other contraindications to CMR examination (arrhythmia, pacemaker, metal implants, claustrophobia, and known or suggested contrast agent allergy to gadolinium).

### Cardiac Magnetic Resonance Imaging

All subjects performed image acquisition on a 3.0-T scanner (Ingenia 3.0 T, Philips, Netherlands) within the first week of pPCI treatment. The patients were supine, and images were obtained under the breath-hold using a digital stream (dS) anterior phased-array surface coil and an integrated dS posterior spine matrix coil. The standardized imaging protocol was implemented according to the current recommendations (9). Modification as a standard imaging protocol, cine images on short axes covering the LV (10–12 slices) were collected after the administration of gadolinium-based contrast agent (0.1 mmol/kg), and delay enhanced images (Late Gadolinium Enhancement; LGE) were obtained after 10–15 min. The parameters were as follows: slice thickness 7 mm; echo time (TE) 1.4 ms; repetition time (TR) 2.8 ms; field of view (FoV) 300 × 300 mm, matrix 280 × 240).

Left ventricular function parameters and myocardial strain were analyzed using CVI (v5.13.5, Circle Vascular Imaging, Canada). The LV endocardial and epicardial borders were automatically traced at end-diastole in three long-axis planes (2, 3, and 4 chambers) and a short-axis plane. To ensure the accuracy, the tracking performance was checked after the automatic strain analysis, and the initial profile was manually adjusted if improper. Two experienced radiologists (with more than 3 years of working experience) independently analyzed all CMR images, and interobserver reproducibility was assessed by performing the same analysis in the randomly generated set of 30 patients (Supplementary Table 1).

**Figure 1:**
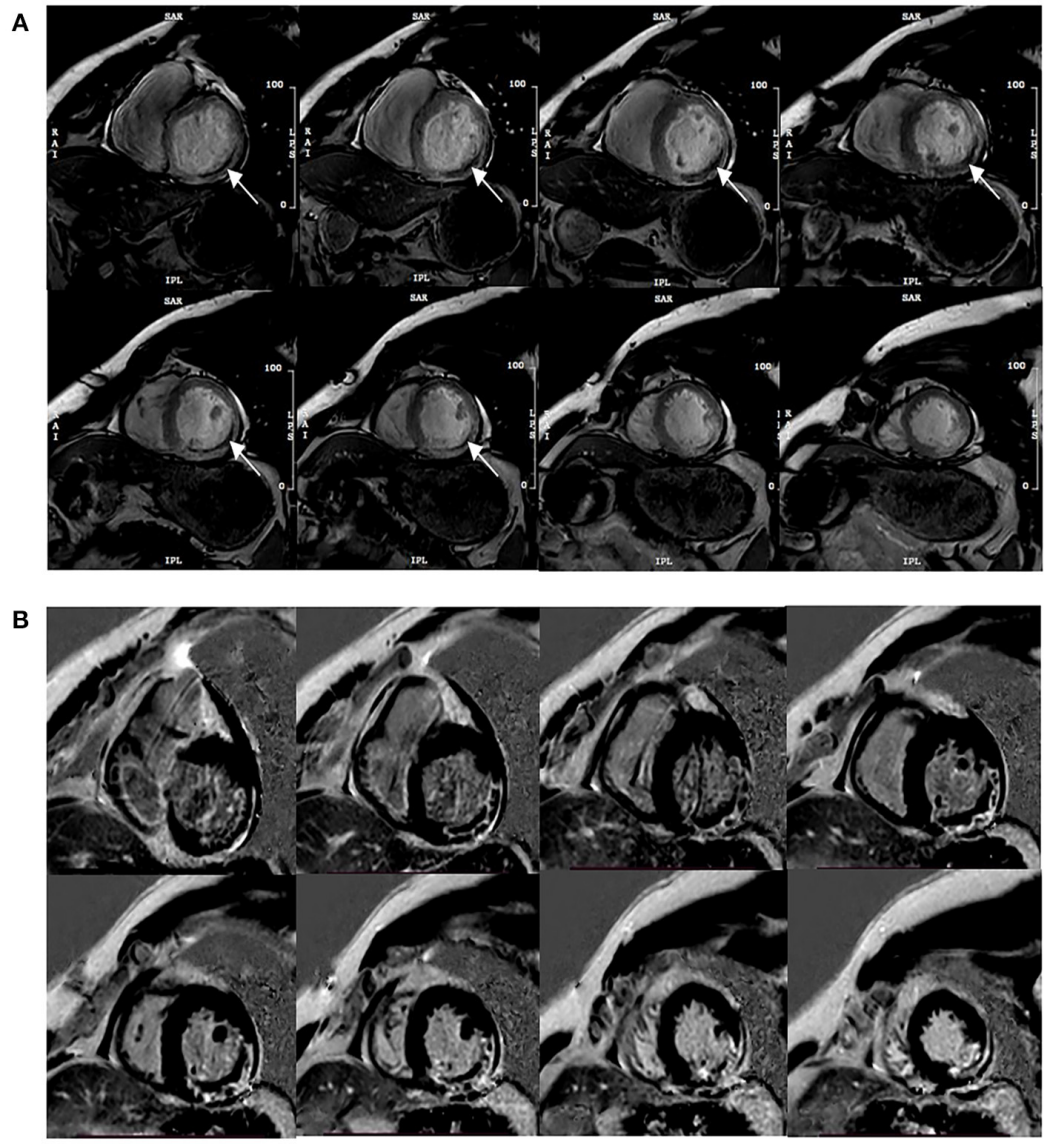
Typical MVO appearance on contrast-enhanced cine (CE-SSFP) imaging at the same slice position during the cardiac cycle **(A)**, and the corresponding LGE images **(B)**. Arrows point to the dark area surrounded by the hyperintensity infarction zone constantly visible and located at the same position within the cardiac wall on each SSFP image during the cardiac cycle.

Functional parameters were assessed on short-axis views, and papillary muscles were assigned to the LV volume, such as LVEF, end-diastolic volume (EDV), end-systolic volume (ESV), and stroke volume (SV). MVO recognition could be achieved during the visually corrected tracking performance without an additional MRI method to make a more reliable assessment. The area at risk (AAR) in CE-cine SSFP images was a visual hyperintensity area compared with the remote myocardium (10). MVO was defined as middle or subendocardial dark areas within AAR. To eliminate low signal artifacts similar to MVO areas, a “true MVO” was defined as a dark area surrounded by the constantly visible hyperintensity zone and located at the same position within the cardiac wall on each SSFP image during the complete cardiac cycle (11) (Figure 1). IS was expressed as a percentage of LV myocardial mass (LVMM), measured from short-axis LGE images (signal intensity > 5 SD of unenhanced remote myocardium) (12), and included MVO. Myocardial strain parameters were obtained from the long-axis cine and short-axis cine to calculate the peak strain parameters in diastole, such as the global peak longitudinal strain (GLS), global peak circumferential strain (GCS), and global peak radial strain (GRS).

### Follow-Up and Endpoint Definition

All patients were followed up *via* telephone and interviewed by a cardiologist after discharge. Major cardiovascular adverse events (MACEs) included all-cause death, myocardial reinfarction, and new congestive HF after discharge. New congestive HF was defined as the first episode of cardiac decompensation that required intravenous diuretic therapy with or without hospital readmission (13).

### Statistical Analyses

Continuous variables are expressed as the mean ± SD or median and interquartile range (IQR) and categorical variables are expressed as the frequency and percentage. The Mann-Whitney U test and the non-paired *t*-test test the differences in continuous and categorical variables between the two groups. Cox regression analyses were performed to reveal the predictive factors of MACE. ROC analyses were applied to evaluate the AUC to predict MACE. Youden index was calculated to evaluate the best cut-off value of the dichotomy of continuous MACE predictors. MACE-free survival was estimated and described using the Kaplan-Meier method, and the log-rank test assessed the differences. *p* < 0.05 was considered statistically significant (SPSS Statistics v26.0).

## Results

### Study Population

In total, 237 patients with acute STEMI who received pPCI treatment for the first time were evaluated and enrolled in this study; 9 (3.7%) patients failed to complete the scan, and 13 (5.4%) patients had poor image quality. Finally, a follow-up analysis was performed in 215 patients (age 58 [IQR: 48–67] years; 19% female). Detailed baseline characteristics are shown in Table 1

**Table 1:** Patient characteristics.

	**All Patients**	**MACE**	**No MACE**	***P***
	***n =* 215**	***n =* 30**	***n =* 185**	
Age, years	58(48–67)	64(56–69)	57(48–66)	0.024
Female, *n*(%)	40(19)	9(30)	31(17)	0.084
Body mass index, kg/m^2^	26 ± 3	26 ± 4	26 ± 3	0.755
Current smoker, *n* (%)	114(53)	13(43)	101(55)	0.252
Diabetes mellitus, *n* (%)	45(21)	5(17)	40(22)	0.536
Hypertension, *n* (%)	101(47)	15(50)	86(47)	0.721
Systolic blood pressure, mmHg	127 ± 19	126 ± 20	127 ± 19	0.747
Diastolic blood pressure, mmHg	80 ± 12	79 ± 13	81 ± 12	0.475
Heart rate on admission, bpm	71 ± 25	76 ± 21	70 ± 25	0.290
Total ischemia time, min	381 ± 59	443 ± 306	370 ± 546	0.294
Door-to-balloon time, min	78 ± 69	64 ± 31	80 ± 73	0.253
Number of affected vessels, *n* (%)				0.666
1	63(29)	10(33)	53(29)	
2	65(30)	7(23)	58(31)	
3	87(40)	13(43)	74(40)	
Culprit lesion, *n* (%)				0.047
LAD	115(53)	21(70)	94(51)	
LCX	29(13)	5(17)	24(13)	
RCA	71(33)	4(13)	67(36)	
TIMI flow pre-pPCI, *n* (%)				0.103
0	147(68)	25(83)	122(66)	
1	3(1)	0(0)	3(2)	
2	37(17)	5(17)	32(17)	
3	28(13)	0(0)	28(15)	
Peak NT-pro BNP, pg/ml	1,580(810–2,839)	2,527(1,674–3,974)	1,400(763–2,615)	0.001
Peak hs-cTnT, ng/l	3,325(1,407–5,831)	4,951(3,476–8,735)	3,087(1,341–5,268)	0.001
LDL cholesterol, mg/dL	2.8 ± 0.9	2.7 ± 0.9	2.8 ± 0.9	0.617

*MACE, major adverse cardiac events; RCA, right coronary artery; LAD, left anterior descending artery; LCX, left circumflex artery; TIMI, thrombolysis in myocardial infarction; NT-proBNP, N terminal pro B type natriuretic peptide; Hs-cTnT, high-sensitivity cardiac troponin T; LDL, low-density lipoprotein*.

### CMR Parameters

Cardiac magnetic resonance imaging scans were performed at a median of 5 [IQR: 4–7] days after PCI, and the standardized imaging protocol time was about 40 [IQR: 34–44] min; however, as a modification, the time of CE-cine was about 27 [IQR: 24–31] min. Table 2 depicts the CMR parameters of all patients. The LVEF was 52 [IQR: 49–57] %, the GLS was −12.0 [IQR: −15.0 to −10.0] %, the GRS was 24 (IQR, 19–30) %, and the GCS was −15 (IQR, −18 to −10) %. The infarct characteristics were IS% 9.3 [IQR: 5.9–15.3] %, and MVO was identified in 109 (51%) patients using CE-cine SSFP imaging and 107 (50%) using LGE imaging.

**Table 2:** CMR parameters of the study population.

	**All Patients**	**MACE**	**No MACE**	***P***
	***n =* 215**	***n =* 30**	***n =* 185**	
EDV/BSA (ml/m^2^)	74(67–85)	83(70–96)	74(66–84)	0.006
ESV/BSA (ml/m^2^)	36(27–46)	45(41–55)	34(27–45)	<0.001
SV/BSA (ml/m^2^)	38(32–45)	33(29–40)	39(33–45)	0.002
LV ejection fraction, %	52(49–57)	49(42–53)	52(50–57)	0.002
CO (L/min)	6.1 ± 7.3	6.1 ± 7.1	6.2 ± 8.5	0.933
CI (L/min/m^2^)	2.7 ± 0.7	2.6 ± 0.7	2.7 ± 0.7	0.287
GRS, %	24.3(19.4–30.3)	20(17–24)	25.1(20.5–30.7)	0.001
GCS, %	−15.0(−17.7 to −12.5)	−12.7(−15.1 to −11.2)	−15.4(−17.8 to −13.0)	<0.001
GLS, %	−12.3(−14.9 to −9.8)	−9.9(−11.9 to −7.00)	−12.5(−15.4 to −10.2)	<0.001
IS, %	9.3(5.9–15.3)	14.3(8.2–25)	8.7(5.3–13.4)	<0.001
MVO, *n* (%)	109(51)	25(83)	84(45)	<0.001

*GCS, left ventricular global circumferential strain; GLS, global longitudinal strain; GRS, global radial strain; EDV, ventricular end-diastolic volume; ESV, ventricular stroke volume; MVO, microvascular obstruction; SV, ventricular stroke volume; BSA, body surface area; CO, cardiac output; IS, infarct size*.

### Clinical Outcome

During a median follow-up time of 13 (QR: 11–24) months, 30 patients (14%) experienced MACE (5 reinfarctions and 25 HF). Compared with patients without MACE, the patients with MACE were older (*p* = 0.024), and most of the culprit's vessels were lamin-associated domains (LADs; *p* = 0.047). In addition, the peaks of high-sensitivity cardiac troponin T (hs-cTnT) and N-terminal pro-B-type natriuretic peptide (NT-proBNP) were higher in patients with MACE (*p* < 0.001).

### CMR Measures and MACEs

The patients in the MACE group had lower LVEF (*p* = 0.002), larger IS (*p* < 0.001), and higher incidence of MVO (*p* < 0.001). GLS, GRS, and GCS were obviously related to MACE (*p* < 0.05). Accordingly, in the subgroup, the GLS and GCS of the MACE group were higher (−10% vs. −13%, *p* < 0.001) (−13% vs. −15%, *p* < 0.001), while the GRS was lower (20% vs. 25, *p* = 0.001).

The univariate and multivariate Cox regression analyses show that (Table 3) the age, culprit vessel, peak hs-cTnT, NT-proBNP, LV EDV index (EDV/BSA), SV index (SV/BSA), LVEF, and all three myocardial strain parameters were predictors of adverse events. However, only GLS, IS, and the presence of MVO were independently associated with MACE in a multivariate Cox analysis model.

**Table 3:** Predictors of MACE in univariable and multivariable Cox regression analysis.

**Variable**	**Univariate**		**Multivariate**	
	**HR (95% CI)**	***P-*value**	**HR (95% CI)**	***P-*value**
Age, years	1.04 (1.003–1.069)	0.035	1.04 (1.000–1.073)	0.047
Female, *n* (%)	1.91 (0.876–4.175)	0.104		
Body mass index, kg/m^2^	0.99 (0.885–1.100)	0.807		
Current smoker, *n* (%)	1.49 (0.727–3.084)	0.273		
Diabetes mellitus, *n* (%)	1.37 (0.525–3.581)	0.520		
Hypertension, *n* (%)	1.14 (0.558–2.333)	0.719		
Culprit lesion, *n* (%)	0.59 (0.365–0.940)	0.027		
TIMI flow pre-pPCI	0.64 (0.413–0.983)	0.420		
Peak NT-pro BNP, pg/ml	1.00 (1.000–1.000)	0.002		
Peak hs-cTnT, ng/l	1.00 (1.000–1.000)	<0.001		
LDL, mg/dL	0.91 (0.604–1.359)	0.633		
EDV/BSA (ml/m^2^)	1.03 (1.012–1.053)	0.002		
ESV/BSA (ml/m^2^)	1.02 (1.009–1.051)	<0.001		
SV/BSA (ml/m^2^)	0.94 (0.905–0.979)	0.002		
EF, %	0.92 (0.873–0.962)	<0.001		
CO (L/min)	1.00 (0.956–1.052)	0.920		
CI (L/min/m^2^)	0.71 (0.400–1.268)	0.248		
GRS, %	0.92 (0.876–0.970)	0.002		
GCS, %	1.17 (1.071–1.272)	<0.001		
GLS, %	1.14 (1.072–1.205)	<0.001	1.08 (1.008–1.165)	0.029
IS, %	1.05 (1.023–1.074)	<0.001	1.03 (1.001–1.063)	0.042
MVO, *n* (%)	4.95 (1.895–12.936)	0.001	3.10 (1.137–8.989)	0.028

*TIMI, thrombolysis in myocardial infarction; NT-proBNP, N terminal pro B type natriuretic peptide; Hs-cTnT, high-sensitivity cardiac troponin T; LDL, low-density lipoprotein; GCS, left ventricular global circumferential strain; GLS, global longitudinal strain; GRS, global radial strain; EDV, ventricular end-diastolic volume; ESV, ventricular stroke volume; MVO, microvascular obstruction; SV, ventricular stroke volume; BSA, body surface area; CO, cardiac output; IS, infarct size*.

**Figure 2:**
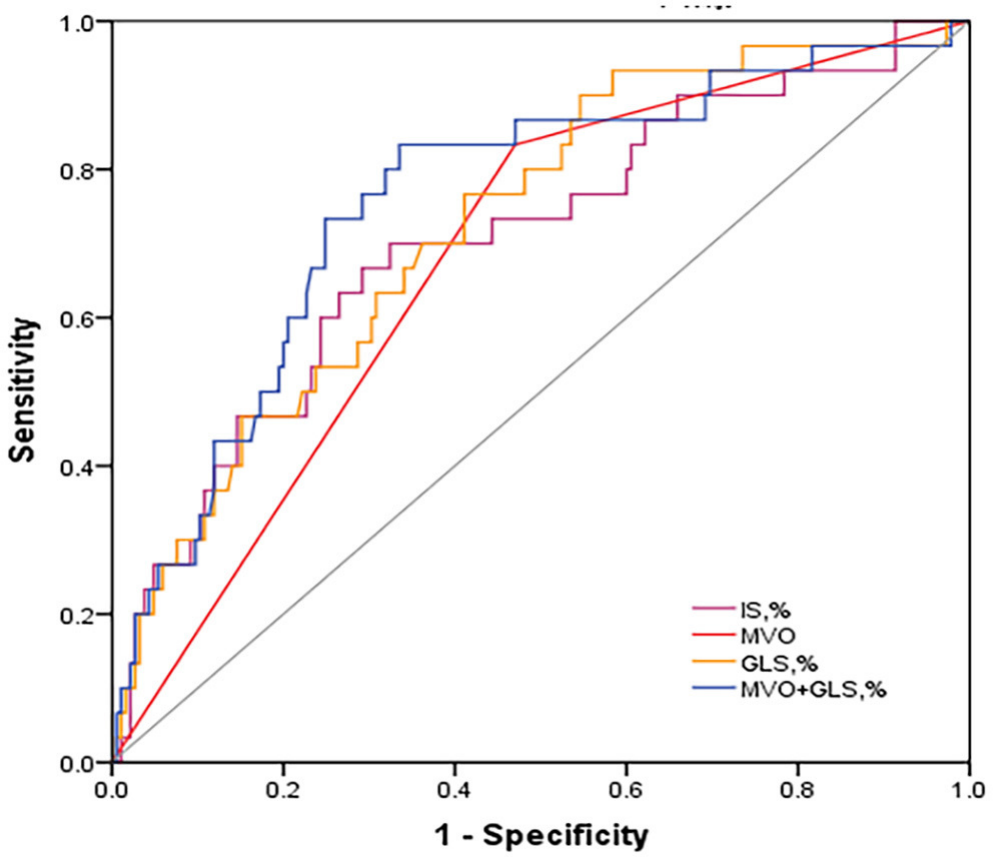
Discriminative prognostic power of MVO, IS, GLS and MVO combined with GLS. ROC curves of them for the prediction of MACE. MVO combined with GLS revealed a significantly higher AUC (0.759, 95% CI: 0.663–0.854, *P* < 0.001) with 83.3% sensitivity and 66.5% specificity. ROC, receiver operating characteristic; AUC, area under the curve; MACE, major adverse cardiac events; GLS, global longitudinal strain; IS, infarct size; MVO, microvascular obstruction.

From the ROC analysis (Figure 2), GLS showed a large AUC in the prediction of MACE (0.73, 95% CI: 0.63–0.82, *p* = 0.001), and the best cut-off was −11.8%, which is better than IS (0.70, 95% CI: 0.60–0.81, *p* < 0.001) and MVO (0.68, 95% CI: 0.58–0.78, *p* < 0.001); However, combining MVO and GLS yielded the largest AUC for the prediction of MACE (AUC = 0.775, 95% CI: 0.727–0.824, *p* < 0.001) and better sensitivity and specificity (83.3%, 66.5%; vs. GLS with 76.7% sensitivity and 58.9% specificity, vs. MVO with 83.3% sensitivity and 53% specificity).

**Figure 3:**
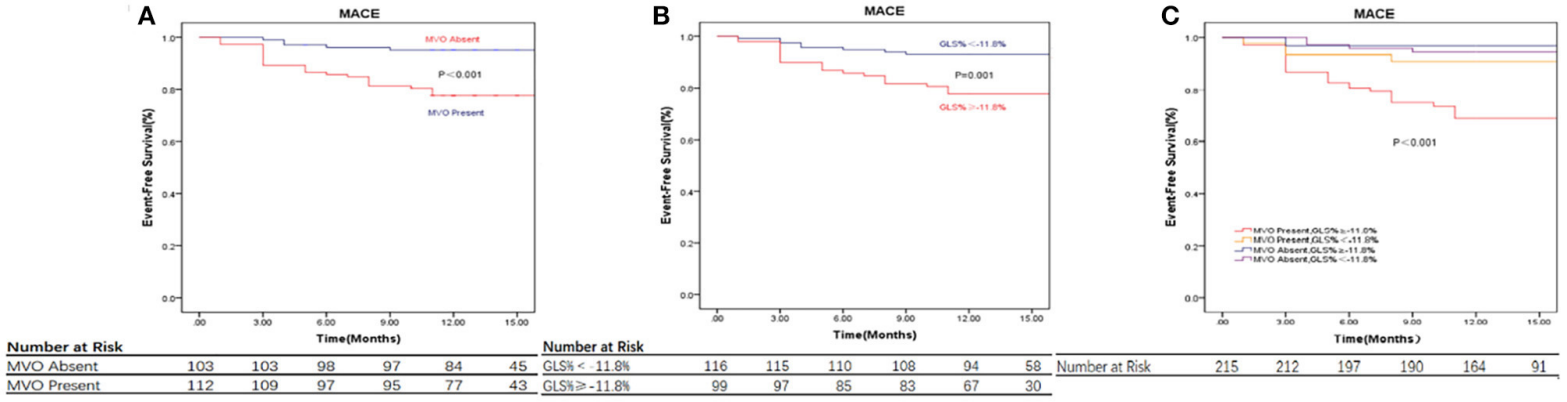
CMR parameters and clinical outcome. **(A)** Displaying the relationship between MVO, Values are Kaplan-Meier estimates in patients with MVO vs. patients without MVO, indicating the time to major adverse cardiovascular events (MACE). **(B)** Displaying the relationship between GLS, Values are Kaplan-Meier estimates in patients with GLS≥ −11.8% vs. < -11.8%, indicating the time to MACE. **(C)** Displaying the relationship between MVO and GLS and Event-Free Survival, Values are Kaplan-Meier estimates in patients with GLS≥ −11.8% vs. < -11.8%, grouped by the presence or absence of MVO, indicating the time to MACE. Patients with GLS≥ −11.8% and MVO had the highest event rates. MACE, major adverse cardiac events; GLS, global longitudinal strain; IS, infarct size; MVO, microvascular obstruction.

Patients were further divided into four groups according to the cut-off value of GLS (−11.8%) and the presence of MVO: MVO present and GLS ≥ −11.8% (*n* = 68); MVO present and GLS < −11.8% (*n* = 44); MVO absent and GLS ≥ −11.8% (*n* = 31); and MVO absent and GLS < −11.8% (*n* = 72). The Kaplan-Meier curves indicate a noticeable difference in cumulative MACE (Figure 3). Patients with impaired strain (GLS ≥ −11.8%) and MVO were prone to events, while the differences among other groups were less pronounced, and the cumulative survival rate free from MACE was GLS < −11.8% in the absence of the MVO group.

## Discussion

This study used CE-SSFP cine images to quickly identify MVO and performed myocardial strain analyses to comprehensively evaluate the prognosis of acute patients with STEMI revascularized by pPCI. The significant findings were as follows: it is reliable to identify MVO through CE-SSFP and can be an option for patients with limited ability to complete the standard CMR protocol. The combination of MVO identification from contrast-enhanced cine and GLS by FT-CMR correlates well with the prognosis of patients with STEMI in terms of MACE, which would be a valuable predictor of MACE and a valuable tool to stratify risk.

Despite successfully reopening the occluded epicardial coronary artery through primary PCI, MVO inevitably occurred in ~50% of patients with STEMI who received pPCI and CMR scans within the time frame (14, 15). In most cases, it was entirely resolved after 8 months. However, a meta-analysis by Hamirani et al. confirmed the intimate relationships of MVO with worse systolic function, increased ventricular volumes, more prominent IS, higher risk of adverse remodeling, and diffused tissue alterations in the non-infarcted myocardium (3, 14, 16–18). MVO is an independent predictor of MACE (hazard ratio [HR] 3.74, 95% CI: 2.21–6.64, *p* ≤ 0.001) (19). This result is consistent with the analysis of Suzanne de Waha, where 1,688 patients underwent CMR within 7 days following STEMI (2) with a median follow-up time of 1 year, and the HR for all-cause mortality was 1.09 (95% CI: 1.01–1.17, *p* = 0.03). MVO is a solid short- and long-term prognostic marker for MACE after STEMI.

Due to its high spatial resolution and tissue composition characteristics, CMR has become the gold standard to quantify myocardial injury non-invasively (20). CMR-related indicators, such as IS (21), AAR (22), the myocardial salvage index (MSI) (23, 24), IMH (25, 26), and MVO (27) are strong predictors of LV remodeling and outcomes in patients with MI. CMR can comprehensively evaluate cardiac function and tissue characteristics in a single examination. Unfortunately, long-term scanning and multiple long breaths holding in patients with MI, especially those who suffer from dyspnoea, are limiting factors in clinical settings (28). It takes at least 40–50 min to complete a comprehensive standard heart procedure (29), and frail patients with recent STEMI may find it difficult.

Given the inefficiency of imaging time and the pursuit of patient comfort and completion, a feasible protocol is required. Cine with b-SSFP is an essential part of each protocol, has the advantages of fast speed, high signal-to-noise ratio, and short breath-hold time; moreover, it provides a detailed analysis of the chamber volumes and cardiac function (mainly EF) with the crucial prognostic value (30). Importantly, cine holds much more detailed information, enabling the assessment of more comprehensive markers of the cardiac function, such as myocardial strain, which may be even more informative for prognostication after STEMI than the standard chamber volumes and EF (6, 31, 32). After contrast medium, shortened exam time and standard b-SSFP imaging can be acquired without loss of accuracy for regional and global ventricular function (33).

Contrast-enhanced-CMR assesses microcirculation in different manners. The first-pass perfusion (FPP) (within the first minute after intravenous injection of the contrast medium) (34), but FPP images have a low signal-to-noise ratio, spatial coverage, and ventricular coverage, which have been criticized as too sensitive because of their changes in most patients with STEMI (35). LGE is the most reliable method to identify and evaluate MVO; nevertheless, the standardized imaging protocol, such as LGE, requires a long time to complete the scan and frequent breath-holding, which was about 40 min in our study, as a modification, the time of CE-cine was about 27 min. Moreover, in the early post-MI phase, the infarct area contains heterogeneous pathology, such as edema, hemorrhage, inflammatory cell infiltration, viable tissue, and dead tissue, and the size of MVO on LGE images is initially larger than repeat evaluations several months later (6). It is challenging to accurately quantify the heterogeneity of small low-signal areas on LGE images, which requires experienced radiologists and much analysis time and is not conducive to widespread clinical application, especially in some non-highly specialized centers. However, MVO can be detected with contrast cine SSFP imaging (11). In our study, MVO was identified in 109 (51%) patients using CE-cine SSFP imaging and 107 (50%) in LGE imaging, comparable to LGE, CE-cine can serve as a backup when LGE imaging is unavailable (10, 11) and can shorten scanning time. Therefore, MVO identification from CE-SSFP can be sufficient for clinical application, and it has a high degree of completion and accuracy, can be an option in patients who are limited in their ability to comply with the demands of a CMR protocol.

Cardiac mechanics are complex and do not enable us to study the spatial organization of myocardial fibers. Strain is the change in length per unit tissue, reflects the myocardial deformation, and is more closely related to cardiomyocyte metabolism and contractility (36), which enables us to study different spatial components of contractile function in the longitudinal strain (LS), circumferential strain (CS), and radial strain (RS) directions (37). The myocardial strain analysis evaluates myocardial deformation throughout the cardiac cycle to determine the global and local LV function (37, 38). FT-derived global longitudinal and CS are predictors of adverse remodeling and poor prognosis in the longer-term post-MI (39–41), especially GLS independent of LVEF and IS. As described by the wavefront phenomenon, the longitudinal myocardial fiber in the subendocardial region appears to be sensitive to ischemia. Irreversible myocardial injury begins under the endocardium and primarily affects the longitudinal contraction, which explains the high sensitivity and prognostic influence of GLS in patients with STEMI. Therefore, GLS is a more comprehensive marker recommended for optimized risk stratification in patients with STEMI. In our study, a GLS of −11.8% was the best cut-off to predict MACE compared to previous analyses (42), combining MVO and GLS indicated a much more significant predictive value (AUC = 0.775, 95% CI: 0.727–0.824, *p* < 0.001) with 83.3% sensitivity and 66.5% specificity. These potential markers of myocardial function and myocardial pathology after STEMI derived from CE-SSFP can be helpful as early markers of subclinical impairment and adverse prognosis.

## Study Limitations

First, as an inherent limitation of cardiac MRI, only stable patients (Killip <3) with first STEMI treated with pPCI were included; hence, our conclusions may not be extrapolated to all patients with STEMI. Second, emerging CMR parameters (including native T1 mapping, extracellular volume, and T2^*^ mapping) were recently recommended for improved risk stratification. We did not perform the measurements because these sequences were unavailable, implemented in subsequent studies. Finally, inconsistencies between commercially available strain assessment software should be considered, where differences between GLS and GCS are acceptable, whereas GRS is not.

## Conclusion

Comprehensive CMR parameters, such as the myocardial function (mostly myocardial strain) and myocardial pathology (mainly MVO), may constitute the most informative methods for patients with STEMI. MVO identification from CE-SSFP combined with myocardial strain can be a valuable predictor of MACE and a helpful tool for risk stratification; it is a quicker and reliable option for patients with STEMI.

## Data Availability Statement

The original contributions presented in the study are included in the article/Supplementary Material, further inquiries can be directed to the corresponding author/s.

## Ethics Statement

The studies involving human participants were reviewed and approved by the Ethics Committee of Affiliated Hospital of Xuzhou Medical University. The patients/participants provided their written informed consent to participate in this study.

## Author Contributions

MZ and YL were significant contributors to the research design, data interpretation, manuscript writing, and modification. YS and LC were involved in data collection and analysis. ZL and YY performed the patient studies. JX performed CMR image acquisition and post-processing analysis. TJ directed the entire research work and corrected the articles. All authors contributed to the article and approved the submitted version.

## Conflict of Interest

The authors declare that the research was conducted in the absence of any commercial or financial relationships that could be construed as a potential conflict of interest.

## Publisher's Note

All claims expressed in this article are solely those of the authors and do not necessarily represent those of their affiliated organizations, or those of the publisher, the editors and the reviewers. Any product that may be evaluated in this article, or claim that may be made by its manufacturer, is not guaranteed or endorsed by the publisher.

## References

[1] Bulluck H Foin N Tan JW Low AF Sezer M Hausenloy DJ. Invasive assessment of the coronary microcirculation in reperfused ST-segment-elevation myocardial infarction patients: where do we stand? Circ Cardiovasc Interv. (2017) 10:e004373. 10.1161/CIRCINTERVENTIONS.116.004373 28242607

[2] de Waha S Patel MR Granger CB Ohman EM Maehara A Eitel I. Relationship between microvascular obstruction and adverse events following primary percutaneous coronary intervention for ST-segment elevation myocardial infarction: an individual patient data pooled analysis from seven randomized trials. Eur Heart J. (2017) 38:3502–10. 10.1093/eurheartj/ehx414 29020248

[3] Reindl M Eitel I Reinstadler SJ. Role of cardiac magnetic resonance to improve risk prediction following acute ST-elevation myocardial infarction. J Clin Med. (2020) 9:1041. 10.3390/jcm9041041 32272692 PMC7231095

[4] Eitel I de Waha S Wohrle J Fuernau G Lurz P Pauschinger M. Comprehensive prognosis assessment by CMR imaginging after ST-segment elevation myocardial infarction. J Am Coll Cardiol. (2014) 64:1217–26. 10.1016/j.jacc.2014.06.1194 25236513

[5] Stiermaier T Jobs A de Waha S Fuernau G Poss J Desch S. Optimized prognosis assessment in ST-segment-elevation myocardial infarction using a cardiac magnetic resonance imaginging risk score. Circ Cardiovasc Imag. 2017 10:e006774. 10.1161/CIRCIMAG.117.006774 29122844

[6] Mangion K McComb C Auger DA Epstein FH Berry C. Magnetic resonance imaginging of myocardial strain after acute ST-segment-elevation myocardial infarction: a systematic review. Circ Cardiovasc Imag. (2017) 10:e006498. 10.1161/CIRCIMAG.117.006498 28733364

[7] Kraigher-Krainer E Shah AM Gupta DK Santos A Claggett B Pieske B. Impaired systolic function by strain imaginging in heart failure with preserved ejection fraction. J Am Coll Cardiol. (2014) 63:447–56. 10.1016/j.jacc.2013.09.052 24184245 PMC7195816

[8] Thygesen K Alpert JS Jaffe AS Chaitman BR Bax JJ Morrow DA. Fourth universal definition of myocardial infarction. Circulation. (2018) 138:e618–e51. 10.1161/CIR.0000000000000617 30571511

[9] Kawel-Boehm N Hetzel SJ Ambale-Venkatesh B Captur G Francois CJ Jerosch-Herold M. Reference ranges (“normal values”) for cardiovascular magnetic resonance (CMR) in adults and children: 2020 update. J Cardiovasc Magn Reson. (2020) 22:87. 10.1186/s12968-020-00683-3 33308262 PMC7734766

[10] Goransson C Ahtarovski KA Kyhl K Lonborg J Nepper-Christensen L Bertelsen L. Assessment of the myocardial area at risk: comparing T2-weighted cardiovascular magnetic resonance imaginging with contrast-enhanced cine (CE-SSFP) imaging-a DANAMI3 substudy. Eur Heart J Cardiovasc Imag. (2019) 20:361–6. 10.1093/ehjci/jey106 30085055

[11] Wuest W Lell M May M Scharf M Schlundt C Achenbach S. Determining microvascular obstruction and infarct size with steady-state free precession imaginging cardiac MRI. PLoS ONE. (2015) 10:e0119788. 10.1371/journal.pone.0119788 25793609 PMC4368429

[12] Bondarenko O Beek AM Hofman MB Kuhl HP Twisk JW van Dockum WG. Standardizing the definition of hyperenhancement in the quantitative assessment of infarct size and myocardial viability using delayed contrast-enhanced CMR. J Cardiovasc Magn Reson. (2005) 7:481–5. 10.1081/JCMR-200053623 15881532

[13] Reindl M Reinstadler SJ Feistritzer HJ Theurl M Basic D Eigler C. Relation of low-density lipoprotein cholesterol with microvascular injury and clinical outcome in revascularized ST-elevation myocardial infarction. J Am Heart Assoc. (2017) 6:e006957. 10.1161/JAHA.117.006957 29018020 PMC5721881

[14] Hamirani YS Wong A Kramer CM Salerno M. Effect of microvascular obstruction and intramyocardial hemorrhage by CMR on LV remodeling and outcomes after myocardial infarction: a systematic review and meta-analysis. JACC Cardiovasc Imag. (2014) 7:940–52. 10.1016/j.jcmg.2014.06.012 25212800 PMC4301583

[15] Galea N Dacquino GM Ammendola RM Coco S Agati L De Luca L. Microvascular obstruction extent predicts major adverse cardiovascular events in patients with acute myocardial infarction and preserved ejection fraction. Eur Radiol. (2019) 29:2369–77. 10.1007/s00330-018-5895-z 30552479

[16] Reinstadler SJ Metzler B Klug G. Microvascular obstruction and diastolic dysfunction after STEMI: An important link? Int J Cardiol. (2020) 301:40–1. 10.1016/j.ijcard.2019.10.051 31732184

[17] Reinstadler SJ Stiermaier T Liebetrau J Fuernau G Eitel C de Waha S. Prognostic significance of remote myocardium alterations assessed by quantitative noncontrast T1 mapping in ST-segment elevation myocardial infarction. JACC Cardiovasc Imag. (2018) 11:411–9. 10.1016/j.jcmg.2017.03.015 28624398

[18] Wong DT Leung MC Richardson JD Puri R Bertaso AG Williams K. Cardiac magnetic resonance derived late microvascular obstruction assessment post ST-segment elevation myocardial infarction is the best predictor of left ventricular function: a comparison of angiographic and cardiac magnetic resonance derived measurements. Int J Cardiovasc Imag. (2012) 28:1971–81. 10.1007/s10554-012-0021-9 22310980

[19] Doherty DJ Sykes R Mangion K Berry C. Predictors of microvascular reperfusion after myocardial infarction. Curr Cardiol Rep. (2021) 23:21. 10.1007/s11886-021-01442-1 33624185 PMC7902326

[20] Sun ZQ Yu TT Ma Y Ma QM Jiao YD He DX. Depression and myocardial injury in ST-segment elevation myocardial infarction: a cardiac magnetic resonance imaginging study. World J Clin Cases. (2020) 8:1232–40. 10.12998/wjcc.v8.i7.1232 32337197 PMC7176617

[21] Wu E Ortiz JT Tejedor P Lee DC Bucciarelli-Ducci C Kansal P. Infarct size by contrast enhanced cardiac magnetic resonance is a stronger predictor of outcomes than left ventricular ejection fraction or end-systolic volume index: prospective cohort study. Heart. (2008) 94:730–6. 10.1136/hrt.2007.122622 18070953

[22] Raman SV Simonetti OP Winner MW 3rd Dickerson JA He X Mazzaferri EL Jr. Cardiac magnetic resonance with edema imaginging identifies myocardium at risk and predicts worse outcome in patients with non-ST-segment elevation acute coronary syndrome. J Am Coll Cardiol. (2010) 55:2480–8. 10.1016/j.jacc.2010.01.047 20510215 PMC3675879

[23] Eitel I Desch S Fuernau G Hildebrand L Gutberlet M Schuler G. Prognostic significance and determinants of myocardial salvage assessed by cardiovascular magnetic resonance in acute reperfused myocardial infarction. J Am Coll Cardiol. (2010) 55:2470–9. 10.1016/j.jacc.2010.01.049 20510214

[24] Eitel I Desch S de Waha S Fuernau G Gutberlet M Schuler G. Long-term prognostic value of myocardial salvage assessed by cardiovascular magnetic resonance in acute reperfused myocardial infarction. Heart. (2011) 97:2038–45. 10.1136/heartjnl-2011-300098 21990384

[25] Wu KC Zerhouni EA Judd RM Lugo-Olivieri CH Barouch LA Schulman SP. Prognostic significance of microvascular obstruction by magnetic resonance imaginging in patients with acute myocardial infarction. Circulation. (1998) 97:765–72. 10.1161/01.CIR.97.8.765 9498540

[26] de Waha S Desch S Eitel I Fuernau G Zachrau J Leuschner A. Impact of early vs. late microvascular obstruction assessed by magnetic resonance imaginging on long-term outcome after ST-elevation myocardial infarction: a comparison with traditional prognostic markers. Eur Heart J. (2010) 31:2660–8. 10.1093/eurheartj/ehq247 20675660

[27] Mather AN Fairbairn TA Ball SG Greenwood JP Plein S. Reperfusion haemorrhage as determined by cardiovascular MRI is a predictor of adverse left ventricular remodelling and markers of late arrhythmic risk. Heart. (2011) 97:453–9. 10.1136/hrt.2010.202028 21051455

[28] Erley J Zieschang V Lapinskas T Demir A Wiesemann S Haass M. A multi-vendor, multi-center study on reproducibility and comparability of fast strain-encoded cardiovascular magnetic resonance imaginging. Int J Cardiovasc Imag. (2020) 36:899–911. 10.1007/s10554-020-01775-y 32056087 PMC7174273

[29] Demirkiran A Everaars H Amier RP Beijnink C Bom MJ Gotte MJW. Cardiovascular magnetic resonance techniques for tissue characterization after acute myocardial injury. Eur Heart J Cardiovasc Imag. (2019) 20:723–34. 10.1093/ehjci/jez094 31131401

[30] Klem I Shah DJ White RD Pennell DJ van Rossum AC Regenfus M. Prognostic value of routine cardiac magnetic resonance assessment of left ventricular ejection fraction and myocardial damage: an international, multicenter study. Circ Cardiovasc Imag. (2011) 4:610–9. 10.1161/CIRCIMAG.111.964965 21911738

[31] Nucifora G Muser D Tioni C Shah R Selvanayagam JB. Prognostic value of myocardial deformation imaginging by cardiac magnetic resonance feature-tracking in patients with a first ST-segment elevation myocardial infarction. Int J Cardiol. (2018) 271:387–91. 10.1016/j.ijcard.2018.05.082 29885827

[32] Claus P Omar AMS Pedrizzetti G Sengupta PP Nagel E. Tissue tracking technology for assessing cardiac mechanics: principles, normal values, and clinical applications. JACC Cardiovasc Imag. (2015) 8:1444–60. 10.1016/j.jcmg.2015.11.001 26699113

[33] Krombach GA Plum T Koos R Hoffmann R Altiok E Kramer NA. Functional cardiac MR imaginging with true fast imaginging with steady-state free precession before and after intravenous injection of contrast medium: comparison of imaginge quality and accuracy. Eur Radiol. (2011) 21:702–11. 10.1007/s00330-010-1969-2 20890762

[34] Mayr A Pedarnig K Klug G Schocke M Pachinger O Jaschke W. Regional functional recovery after acute myocardial infarction: a cardiac magnetic resonance long-term study. Int J Cardiovasc Imag. (2012) 28:1445–53. 10.1007/s10554-011-9951-x 21964639

[35] Rios-Navarro C Marcos-Garces V Bayes-Genis A Husser O Nunez J Bodi V. Microvascular obstruction in ST-segment elevation myocardial infarction: looking back to move forward. Focus on CMR. J Clin Med. (2019) 8:1805. 10.3390/jcm8111805 31661823 PMC6912395

[36] Sengupta PP Narula J. Cardiac strain as a universal biomarker: interpreting the sounds of uneasy heart muscle cells. JACC Cardiovasc Imag. (2014) 7:534–6. 10.1016/j.jcmg.2014.04.001 24831219

[37] Amzulescu MS De Craene M Langet H Pasquet A Vancraeynest D Pouleur AC. Myocardial strain imaging: review of general principles, validation, and sources of discrepancies. Eur Heart J Cardiovasc Imag. (2019) 20:605–19. 10.1093/ehjci/jez041 30903139 PMC6529912

[38] Smiseth OA Torp H Opdahl A Haugaa KH Urheim S. Myocardial strain imaging: how useful is it in clinical decision making? Eur Heart J. (2016) 37:1196–207. 10.1093/eurheartj/ehv529 26508168 PMC4830908

[39] Holzknecht M Reindl M Tiller C Reinstadler SJ Lechner I Pamminger M. Global longitudinal strain improves risk assessment after ST-segment elevation myocardial infarction: a comparative prognostic evaluation of left ventricular functional parameters. Clin Res Cardiol. (2021). 10.1016/S0735-1097(21)02669-3 33884479 PMC8484167

[40] Mangion K Carrick D Carberry J Mahrous A McComb C Oldroyd KG. Circumferential strain predicts major adverse cardiovascular events following an acute ST-segment-elevation myocardial infarction. Radiology. (2019) 290:329–37. 10.1148/radiol.2018181253 30457480

[41] Podlesnikar T Pizarro G Fernandez-Jimenez R Montero-Cabezas JM Sanchez-Gonzalez J Bucciarelli-Ducci C. Five-year outcomes and prognostic value of feature-tracking cardiovascular magnetic resonance in patients receiving early prereperfusion metoprolol in acute myocardial infarction. Am J Cardiol. (2020) 133:39–47. 10.1016/j.amjcard.2020.07.037 32819681

[42] Gavara J Rodriguez-Palomares JF Valente F Monmeneu JV Lopez-Lereu MP Bonanad C. Prognostic value of strain by tissue tracking cardiac magnetic resonance after ST-segment elevation myocardial infarction. JACC Cardiovasc Imag. (2018) 11:1448–57. 10.1016/j.jcmg.2017.09.017 29248649

