# Supplementary material for: Value of Fast MVO Identification From Contrast-Enhanced Cine (CE-SSFP) Combined With Myocardial Strain in Predicting Adverse Events in Patients After ST-Elevation Myocardial Infarction
Source: Front Cardiovasc Med. 2022 Feb 21;8:804020. doi: 10.3389/fcvm.2021.804020 (PMC8900720; doi:10.3389/fcvm.2021.804020)
Supplement: Supplementary file 1 [file Table_1.doc]

| Strain Parameters | ICC | ICC（95%） |
| --- | --- | --- |
| GLS | 0.938 | 0.908-0.958 |
| GCS | 0.931 | 0.857-0.962 |
| GRS | 0.897 | 0.759-0.947 |

**Supplementary Table 1|** ICC of LV Global Myocardium Strain

GCS, global circumferential strain; GLS, global longitudinal strain; GRS, global radial strain,
